# Practical Remarks on Blood-Letting

**Published:** 1866-03

**Authors:** D. B. Trimble

**Affiliations:** Chicago, Ill.


### CHICAGO MEDICAL EXAMINER.

N. S. DAVIS, M.D., EDITOR.

VOL. VII. MARCH, 1866. NO. 3.

### Original Contributions.

### ARTICLE IX.

### PRACTICAL REMARKS ON BLOOD-LETTING.

BY D. B. TRIMBLE, M.D., of Chicago, Ill.

As certain medicines have, at one period, obtained great celebrity in the cure of diseases, and, after a time, have been treated with partial or total neglect, either justly or without cause; so has it been with blood-letting, to a certain, and probably to an unjust, extent. That, in the hands of the profession, a generation or two past, it was used to an injudicious and injurious degree, there can be but little doubt; but the danger of falling into the opposite extreme, and neglecting a remedy so potent, and oftentimes so beneficial, is, I think, imminent. Therefore, as one of the most powerful means we possess of controlling disease, blood-letting should maintain a prominent place in the list of remedies at the disposal of the physician for the amelioration or cure of "the ills that flesh is heir to." That it is too much neglected by the younger members of the profession, at least, of the present day, I think there is no doubt; and it has not been long since one of them, of considerable practice in this community, (and of which he is well deserving,) told me that he had not only never bled any one, but that he had never seen any one bled! With this impression, and with the hope of directing this portion of the profession to its claims upon their attention, I have collected and condensed, from numerous sources, the views and experience of authors and practitioners respecting its employment in the various diseases in which it has been used, and have, also, occasionally given my own.

Some general circumstances should be taken into consideration in enabling us to determine the propriety of blood-letting. If the constitution is vigorous and tending to plethora, it will, of course, bear it much better than if feeble or anæmic. Age, also, should be considered; youth, or middle life, bearing it better than either of the extremes of infancy or senility. It is better borne by the sanguine than by the nervous or phlegmatic temperaments; by a person of temperate, than by one of intemperate habits. Climate and seasons should also influence, as it can be more freely resorted to in cold climates and seasons than in the warm. In the early stages of disease, as a general rule, it is more beneficial than in the later. In all that class of diseases in which the circulation is mainly affected, it is especially beneficial: as in plethoras; vascular irritations; inflammations; fevers; congestions, or sanguineous determinations; hemorrhages; or morbidly increased secretions or effusions.

In this paper, I shall briefly consider those diseases of the *nervous system* in which blood-letting is recommended.

In all accidental injuries of the brain or spinal marrow, blood-letting is frequently beneficial. In fracture of the cranium, concussion or compression of the brain, and hernia cerebri, venesection is required in many cases; and in all, except the last, arteriotomy may be necessary. In all of them, and in fractures or wounds of the vertebræ, local bleeding is beneficial, and venesection is sometimes required in the injuries of the spine and spinal marrow.

In *fracture* of the *cranium*, unaccompanied by depression of the bone, or by inflammatory symptoms, bleeding may sometimes be dispensed with; but, where these conditions make it necessary, the temporal artery should be opened, or a free bleeding from the arm resorted to.

*Concussion.*—In the first stage, or that of depression, bleeding should be avoided; but when reaction takes place, then bleeding freely from the temporal artery or arm is requisite, one or two bleedings will generally suffice, and, if necessary, local bleeding may follow.

In *fracture*, or in *concussion* of the *spine*, where inflammation supervenes, leeches or cups should be applied along its track.

In all the phlegmasiæ of the brain, spinal marrow, and nerves, viz.:—in meningitis; cerebritis; tuberculous meningitis; cerebro-spinal meningitis (acute or epidemic); spinal meningitis; myelitis; or neuritis, both general and local bleeding are mostly indicated. In acute meningitis only, however, is arteriotomy proper; and, in this disease, when caused by hypertrophy of the left ventricle of the heart, bleeding should be more cautiously practised. In all these affections, general bleeding should be used the first few days, or until the severity of the symptoms abate, when cups or leeches may be substituted, until depletion is no more required. In all these affections, except cerebritis, we must be chiefly guided by the condition of the pulse, to determine the amount of blood to be taken: when the pulse becomes softer and slower, the bleeding to be checked, to be renewed when the exacerbation returns. But in *cerebritis*, the pulse is often, if not generally, small and corded, and the effect upon the brain, and system generally, must chiefly guide us as to the quantity drawn. If the pulse, when small and corded, rises under the operation, and becomes fuller and softer, it will, probably, be well borne. Frequently in this disease, and always in *tuberculous meningitis*, it is only in the *early stages* that venesection should be practised; to be followed, if requisite, by the frequent but moderate application of cups or leeches. In acute cerebro-spinal meningitis, it is equally called for; and even in the epidemic variety, or that improperly termed "spotted fever," it has been recommended by some authors; and I cannot but be impressed with the propriety of resorting to it in many of the cases that occur, judging by the symptoms related by those who have reported cases. It is true that I can give no experience regarding the treatment of this formidable malady, as it has never been my fortune, or misfortune, to see a case; yet, it is my belief, notwithstanding the strong terms in which it has been denounced by some authorities, that this powerful agent is too much neglected, or too inefficiently used. When we take into consideration the symptoms—the disease being often ushered in with a chill; followed by severe headache; pains in the neck, spine, limbs; sensitiveness to light and sound; flushed, or turgid countenance; cramps, or spasm of muscles; opisthotonos; hot skin; great thirst; active febrile excitement; pulse, though sometimes depressed, often full and frequent; delirium; stupor; convulsions; coma; the temptation to me would be very strong to use the lancet, notwithstanding the symptoms of debility that eventually, and often speedily, supervene. It is true that, in many cases (as reported,) these symptoms, or the majority of them, do not occur, but a state of extreme debility or collapse prevails from the first; in such cases, depletion would certainly be improper. Only in the earliest stages of those with active inflammatory symptoms, probably, would bleeding be admissable, but the congested, and probably inflamed, condition of the brain and spinal marrow, strongly indicate its use in this stage. The rapid and overpowering influence of the disease, probably, soon induces the low or asthenic condition that supervenes, and if the inflammatory condition could be relieved, this result might not take place. I think these views may receive some support from a number of cases reported by Dr. C. G. PAGE, in the EXAMINER for October, 1865. He reports 19 cases, of which 3 recovered, and 16 died. They were all cases of males, generally in the full vigor of youth, "strong and healthy," and, in most of them, the symptoms before enumerated were present. In several of them the treatment is not mentioned, but in those where it was related, leeching was practised but once, and that with partial relief, and venesection not at all, and the natural inference is, that it was not practised in any case. As in cerebritis, innervation is often so overpowered by the condition of the brain as to depress the circulation, thereby causing a contracted, if not feeble, condition of the pulse, and, sometimes, coolness of the extremities, it is fair to infer that a violent attack of cerebro-spinal meningitis may cause similar phenomena; and if in cerebritis the pulse often becomes fuller, or even firmer, after or during the abstraction of blood, so might, probably, a similar effect be produced by the same means in this affection. I present these views with diffidence, as they are not the result of experience. I know that most authorities regard this as essentially a blood disease; that there is a septic condition of the blood, with local inflammation of the base of the brain; or, that there is a dyscrasia in which the serum is rapidly separated from the crassamentum of the blood, producing serous apoplexy, or convulsions, or asphyxia, etc., according to the part of the body in which the infiltration takes place. All modes of treatment appear to have about equally succeeded (or failed,) and, therefore, basing my treatment upon the semeiology, I should be inclined to practice depletion in the early stages of those cases exhibiting active inflammatory symptoms, speedily following it with stimulants and tonics.

In neuritis, venesection, if the pulse is full and strong, and leeching along the course of the inflamed nerve is recommended.

In the *organic affections* of the brain and nervous system, bleeding has a beneficial influence. In hypertrophy, atrophy, or tumors of the brain, or spinal marrow, both general and local blooding is sometimes called for; in abscesses of the brain, local bleeding only. In all this class of cases, venesection, when requisite, should be used with much more caution than in the preceding class; and in most cases, probably in all those of atrophy, as well as abscesses, should be confined to local bleeding. In the cerebral diseases, leeching or cupping the temples or nucha; and in the spine, over the affected part, would be the indications.

In sanguineous determinations, or congestion of brain or spinal marrow, bleeding, in some form, is very often required. In this class may be mentioned apoplexy; epilepsy; vascular, or passive congestion of brain; delirium ebrietatis; convulsions, and coma.

In *apoplexy*, general bleeding is usually indicated. In very urgent cases, arteriotomy may be preferable, but generally venesection is sufficient. Though bleeding is generally requisite in apoplexy, it should not be indiscriminately practised, but the age and constitution of the patient, his general habits and health, and the causes producing the attack, should all be taken into consideration. Yet, I do not agree with some authorities, that we should be guided by the condition of the pulse alone, or always, in deciding upon the propriety of blood-letting. If the pulse is full and strong, authors advise bleeding, "but if small and feeble, there is no advantage to be derived from the further loss of blood at the commencement of the treatment." So says that eminent (and generally excellent and reliable) authority, Dr. Wood, in his work on *Practice*, vol. ii., pp. 68, 69. But, as the attack is caused by hemorrhage or congestion, and the symptoms from either cause are generally undistinguishable, how are we to decide whether the compression from congestion does not still exist, thus, in a great measure overpowering innervation, and lessening the heart's action, and, consequently, the pulsation of the arteries? This is the view I took of a case some years ago, and I think the result justified it:—

One Sunday morning, about 10 o'clock, I was hurriedly summoned to one of my patients, a large-framed lady, of full habit, and upwards of 60 years old, several of whose family had died of apoplexy. Just as she was about to enter a place of worship, she fell, and was insensible. She was carried into a neighboring house, and, when I arrived, I found her sister, three physicians, and a number of her friends surrounding her. She was reclining on a sofa, with her feet in a hot mustard bath, and cold applications to the head. Her face (usually very florid) was pallid, but swollen; foam passing from her lips; her pulse *barely perceptible.* Her sister (a lady of great intelligence) earnestly besought me to do something for her relief; the other gentlemen probably fearing to advise blood-letting in the aspect of the case. I saw that death must speedily ensue if she was not relieved, and believing that hemorrhage might not yet have taken place, and that innervation was depressed by the congestion and compression of the brain, I laid my views before the three medical gentlemen, and received their full approbation to resort to venesection; and, with the concurrence of the sister, to whom I explained, in few words, the case and its possible unfavorable result, I opened a vein in the arm, with one of the physicians with his fingers on one wrist, while I did the same with the pulse of the other. In a short time, there was an increase in the frequency and fulness of the pulse, and I then opened a vein in the other arm, and after the abstraction of probably twenty ounces of blood, and considerable reaction of the system, as shown by the improved condition of the pulse, the increased warmth of the skin, less dysp-nœa, decreased stertor, etc., I had strong hopes of the recovery of my patient. In the evening she spoke, but not rationally; the next day she would answer questions, but in a confused manner until the evening, when she aroused to full consciousness,and four days from her attack was taken home, when she recovered her usual health. Two years before, she had had symptoms of an attack, which I warded off chiefly by a full bleeding. About two years after the second attack, and after my removal from the place, she died from the same disease. Now, if we had been guided by the pulse alone, as advised by Dr. WOOD, who says, (p. 669,) "I would *repeat*, that the practitioner should be guided by the *strength* of the pulse, whether he shall bleed or not," and *not* have bled this patient, death must have inevitably and speedily occurred; and I think that the prompt and perfect relief given shows that the theory of depressed innervation from congestion, was, in this case at least, correct. On the other hand, I have had cases where the pulse was full and strong, the face red and turgid, and all the indications where bleeding is recommended, and who have, nevertheless, succumbed to the disease, notwithstanding free depletion; so that the pulse cannot always be taken as the guide for this practice.

In *epilepsy*, bleeding should be more cautiously used than in apoplexy, and should depend more upon the etiology than in the former disease. If preceded by meningitis, cerebritis, or cerebral congestion; or from injuries to the cranium, causing compression of the brain; or by a rich or plethoric condition of the blood, especially if threatening apoplexy, venesection should be practised during the paroxysm; and if coma remains after the convulsive efforts cease, a repetition of the bleeding, or leeches or cups to the nucha or temples. The majority of cases, especially those of long standing, do not require it, and in many it would do much injury. Perhaps it would not be irrelevant here to remark that, in my early practice, I had an interesting case of epilepsy, the only *apparent* cause for which was thwarted affection. It was in a young lady, about 20 years old, of full medium height, and robust form and health. Her paroxysms were very violent, and bleeding and other remedies did not cure, though they ameliorated the severity of her disease. They did not recur very often, but were increasing in frequency. She was engaged to a young man, who was employed as supercargo in a vessel that sailed between our ports and the East; and, chiefly, on account of his seafaring life, her mother strenuously opposed her marriage. Connecting the period of the first appearance of the disease with that of her disappointment, and being unable to trace the cause to anything but the restraint she was imposing, from a sense of duty, on her strong will and desires, I thought it might possibly be that if this pressure (which *bleeding* could not remove) was taken off her brain and *heart*, she might probably recover; and, sympathizing with her in her trouble, I felt justified in stating to her mother, with considerable force and urgency, that I believed her daughter's convulsions were caused by the state of her feelings, and *her* opposition to her earnest and unsubdued affection. She was thus infied to reluctantly, to withdraw her opposition, and I was gratiduced, find that for three or four years (after that time I lost sight of them) subsequent to her marriage she had no return of them, and that she was blessed with a healthy child and a good husband. In her case, I have no doubt that the relief to her mental depression, and the consummation of her marriage, effected the cure; yet, taking her sanguine temperament, her tendency to plethora, and the severity of the convulsions into consideration, they might have proved fatal, had she not have been relieved by bleeding.

In *active* or *vascular congestion* of the brain, though not amounting to apoplexy, bleeding is generally required. If not subdued by venesection, then cupping or leeching the temples or nucha should be practised.

In *passive congestion*, or that caused by mechanical means, it is seldom requisite; where it is, local bleeding is generally sufficient.

*Delirium ebrietatis* is another disease in which the loss of blood may relieve unpleasant symptoms. I do not mean that condition of the system brought on in the habitually intemperate by abstaining from their accustomed stimuli, but the state caused by the stimulation of alcoholic liquors, in those not in the *habit* of intemperate drinking. In persons of a full habit, or sanguine temperament, an approach to congestion of the brain, or to meningitis, is sometimes produced by the too free use of liquors, when it becomes necessary to bleed.

In *delirium tremens*, it is, probably, never requisite; though there is sometimes a congested condition of the brain preceding the *tremors*, in which local depletion is required.

*Coma*, though but a symptom, or the effect of disease, may, from its importance, be treated of in this connection. If produced by congestion, or inflammation of the brain or its meninges, or by mechanical pressure, venesection, leeching, or cupping would be appropriate; but if from an anæmic state of the system, or from the prostration of fevers or narcotic poisons, it would be injurious. If the pulse is feeble, the skin cool, and the pupils dilated, bleeding can seldom or never be proper.

In the *convulsions* of children, connected with a full and frequent pulse, a flushed and swollen countenance, and hot skin, bleeding, either general or local, will frequently be required in the paroxysm; and if coma remains long after the paroxysm ceases, it may be repeated during the intermission. But a large number of cases will do well without it.

In *puerperal convulsions*, which are *generally* nearly allied to apoplexy, prompt and efficient venesection is called for, and, in some cases, even arteriotomy may be resorted to. Not only does bleeding unload the congested brain, by lessening the quantity of blood in the body, but, in cases where delivery has not been accomplished, (and this is usually the case,) by its relaxing effect on the system, it causes the os uteri to dilate, and thus facilitates delivery. The pressure on the nervous system and on the circulation being thus removed, the chief cause of the convulsions ceases.

To the *functional* diseases of the brain and nervous system it is less applicable than to any of the classes mentioned, and in most of them may be dispensed with; and, when necessary, local bleeding is generally sufficient. In catalepsy, hysteria, nervous irritation, and cephalalgia venesection may be required occasionally, and with ecstacy, stupor, or vertigo may, more frequently, be treated by local bleeding.

*Catalepsy.*—In this rare disease, the pulse must guide us: if full and tense, venesection may be practised moderately, and cups or leeches applied to the temples or back of the neck.

In *hysterical convulsions*, where the pulse is full and strong, and the face flushed and turgid, blood may be taken from the arm. The causes of hysteria must, in a great degree, guide our treatment; but where there are local determinations of blood to any part, or where there is tenderness and irritation of the spine, local bleeding is often very beneficial.

In *nervous irritation*, when connected with a congested condition of any part of the nervous system, moderate *general* bleeding, or local bleeding may be advised; but it should be with great care, as the majority of cases do not call for it, and in many it would be very injurious.

Though *cephalalgia*, like coma, is only a symptom of disease, and not the pathological condition itself, yet is of so frequent occurrence as to be a subject of interest, and its treatment of much importance. Though no doubt always symptomatic, yet there are cases, the etiology of which cannot be decided, and they are therefore termed idiopathic, for want of a better knowledge of the disease. In cases where there is determination of blood to the brain, or where the pulse is full, tense, or frequent, the head or skin hot, the eyes congested, or a secretion of tears, bleeding, both local and general, may be required, and will often speedily relieve. Even in anæmic cases, there may be a congested condition of the brain from the impaired vigor of circulation; and I have known great relief obtained, in such cases, by the application of a few leeches to the temples. It is probable that in persons predisposed to plethora, a congested condition of the portal circulation is often the cause of severe headache, and that a free evacuation of dark, almost grumous, bile will relieve it. This I have a few times experienced in my own person, and I cannot account for the relief obtained, except on this supposition. In headache from this cause, bleeding may sometimes be necessary.

In ecstacy, stupor, or vertigo, local bleeding may often relieve the symptoms and prevent the accession of more serious diseases, of which they are generally the forerunners.

In some of those diseases of the nervous system, where one of the chief symptoms is deranged motion, bleeding, general and local, is sometimes necessary; especially in chorea, paralysis, and tetanus.

In the cases of *chorea* that have come under my care, I have never found it requsite to bleed, either generally or locally; and as it generally occurs in the young and feeble, and is often connected with an anæmic condition of the system, it will seldom be necessary, indeed, will generally be injurious to deplete. But there are cases in which local, and occasionally general, bleeding may have a beneficial effect; as, for instance, where there is a tendency to congestion of the brain, as indicated by a full and tense pulse, severe headache, and flushed features, leeches to the temples or moderate venesection should be resorted to; or if there is tendernets of the spine, cups or leeches should be applied to the affected part.

In *paralysis*, if caused by meningitis, cerebritis, apoplexy, congestion of brain, spinal meningitis, or myelitis, venesection, or local depletion, or both, are generally necessary in the early stages of the disease, but inapplicable at later periods, or when it has taken on the chronic form.

In *tetanus*, where there are symptoms of cerebral or spinal inflammation, as shown by fever, full and frequent pulse, headache, tenderness of spine, and numbness of lower extremities, venesection and cupping over the spinal column should be practised; yet, many, perhaps most, cases of locked-jaw are produced by other causes, as punctured wounds, etc., in which there is no inflammatory condition of the nervous centres, and in which it is not required.

In *hydrophobia*, bleeding has, in some cases, been practised to, and with a view to, a fatal effect; but as to any *other* result, in respect to arresting or retarding the disease, we have not, that I am aware of, any reliable evidence. In regard to the moral or legal *right* that physicians have, to pursue such a practice, I think there can be no question. Their province is to *cure*, not to *kill*, and if they cannot effect the one result, they have no right to perpetrate the other. As well might we terminate the sufferings, by death, of our patients in any other terrible and generally hopeless disease, as in this.

There is but one other class of diseases of the nervous system to be treated of in this connection, and that is, those of deranged intellection.

In *insanity*, which comprises in its meaning the whole series of deranged intellect, general and local depletion is often called for; though, on this subject, authorities of great weight differ. In all cases of mania produced by cerebral inflammation, both theory and facts warrant us in employing venesection and local bleeding, promptly and freely. If caused by chronic phrenitis, or the puerperal state, more caution should be observed in the use of this measure, but it should not be neglected. If, however, it is to benefit the patient, it should be resorted to in the early stages of his disease; and though it may be serviceable even after insanity has been long confirmed, it is but seldom requisite. If in cases of insanity that have been a month or two under treatment there is not a marked amendment of the symptoms, the best practice the private physician can resort to, is to send his patient to a good asylum for the insane. This has been my rule, and I have not regretted it; for though one or two are yet lingering in hospitals, that were sent there fifteen years ago, by my advice, yet others have been released—some partially, some wholly, cured; and in all cases, they have been more comfortable and better treated, professionally, than it would be possible for them to be at home, and by private practice.

I have thus given a sketch of the diseases of the nervous system in which this potent agent should, or may be used; and I scarcely need remark, that there are so many circumstances attending each individual case of disease, beside its *name*, to influence the decision of the practitioner in the treatment of his patients, that it is difficult to make any rule universally applicable, and on his knowledge and judgment must he alone depend at the bedside of the sick. Yet, the young and inexperienced physician may often be relieved of anxious and perplexing doubts, if he can avail himself of the opinions or experience of others; and, with this object in view, I have crudely and briefly treated of a portion of those diseases that may be benefited or relieved by this one of the agencies we possess, and which, if *properly* employed, is scarcely excelled in importance by any other. The subject will be resumed.

*Chicago, February,* 1866.

